# Identification of SNPs Associated with Somatic Cell Score in Candidate Genes in Italian Holstein Friesian Bulls

**DOI:** 10.3390/ani11020366

**Published:** 2021-02-01

**Authors:** Riccardo Moretti, Dominga Soglia, Stefania Chessa, Stefano Sartore, Raffaella Finocchiaro, Roberto Rasero, Paola Sacchi

**Affiliations:** 1 Department of Veterinary Science, University of Turin, 10095 Turin, Italy; riccardo.moretti@unito.it (R.M.); dominga.soglia@unito.it (D.S.); stefano.sartore@unito.it (S.S.); roberto.rasero@unito.it (R.R.); paola.sacchi@unito.it (P.S.); 2 Associazione Nazionale Allevatori Razza Frisona e Jersey Italiana—ANAFIJ, 26100 Cremona, Italy; raffaellafinocchiaro@anafi.it

**Keywords:** Holstein Friesian cattle, mastitis resistance, candidate genes, SNP selection, next-generation sequencing

## Abstract

**Simple Summary:**

Mastitis is a worldwide diffused disease usually treated with an excessive use of antibiotics. Therefore, antimicrobial resistance is an important issue to be addressed by scientists. One of the possible solutions to decrease the use of drugs is genetic selection of resistant animals, that is, individuals that can be more resistant to mastitis. In our survey we analyzed Single Nucleotide Polymorphisms (SNPs) in genes known to be involved in both infection resistance and immune system activity. We found a group of SNPs that can be associated to mastitis related phenotypes (namely SCS) and that can be used for selecting resistant animals. An efficient selection is able to improve both animal welfare and quality and safety of animal products

**Abstract:**

Mastitis is an infectious disease affecting the mammary gland, leading to inflammatory reactions and to heavy economic losses due to milk production decrease. One possible way to tackle the antimicrobial resistance issue stemming from antimicrobial therapy is to select animals with a genetic resistance to this disease. Therefore, aim of this study was to analyze the genetic variability of the SNPs found in candidate genes related to mastitis resistance in Holstein Friesian bulls. Target regions were amplified, sequenced by Next-Generation Sequencing technology on the Illumina^®^ MiSeq, and then analyzed to find correlation with mastitis related phenotypes in 95 Italian Holstein bulls chosen with the aid of a selective genotyping approach. On a total of 557 detected mutations, 61 showed different genotype distribution in the tails of the deregressed EBVs for SCS and 15 were identified as significantly associated with the phenotype using two different approaches. The significant SNPs were identified in intergenic or intronic regions of six genes, known to be key components in the immune system (namely *CXCR1*, *DCK*, *NOD2*, *MBL2*, *MBL1* and *M-SAA3.2*). These SNPs could be considered as candidates for a future genetic selection for mastitis resistance, although further studies are required to assess their presence in other dairy cattle breeds and their possible negative correlation with other traits.

## 1. Introduction

Mastitis is an infectious disease that affects the mammary gland of cattle, leading to an inflammatory reaction and therefore to negative economic consequences due to a marked decrease in milk production [1]. This infection is usually caused by microorganisms penetrating the mammary gland via teat canal [2]: pathogens can be transmitted either between cows (e.g., *Staphylococcus aureus*) or picked up from the environment (e.g., *Escherichia coli*) [3]. Bovine mastitis is considered as one of the costliest diseases affecting dairy cattle worldwide, with antimicrobial therapy representing the major impact on sustained costs [4]. Furthermore, given the high mastitis frequency worldwide, the subsequent antibiotic use in dairy cows is under constant control due to its association with antimicrobial resistance increase [5].

One possible way to tackle the antimicrobial resistance issue is to select animal with a higher genetic resistance to this disease [6]. Starting from the 50’s, along with its genetic relationship with additional infection-related phenotypes, the possible inheritance of genetic resistance to mastitis was studied through the years [7,8]. However, the first traits to be selected, like mammary gland characteristics and somatic cell count (SCC), revealed to have low to moderate heritability [9]. Therefore, new genetic approaches to find markers able to allow a faster and more accurate selection are requested, with two potential available candidates: genome scanning and single nucleotide polymorphisms (SNPs) in candidate genes [6]. A holistic approach such as genome scanning and/or a Genome Wide Association Study (GWAS) requires a large effect and/or a large number of animals to detect loci associated with traits of interest, while complex diseases are determined by many loci or genes with a small or almost negligible effect [10]. Therefore, the SNP approach in candidate genes involved in organism recognition, leukocyte recruitment, pathogen elimination and resolution seem to be more direct and reliable.

At first, we selected nine genes that are all involved in the immune response to mastitis infection according to literature. Pentraxin 3 (*PTX3*) gene maps on *Bos taurus* autosome 1 (BTA1) and its encoded 45 kDalton glycosylated protein is expressed by mononuclear phagocytes, dendritic and endothelial cells in response to primary inflammatory signals, due to complement activation and pathogen recognition [11]. Chemokine (C-X-C motif) receptors 1 and 2 (*CXCR1* and *CXCR2*, respectively) are paralogous genes coding for major proinflammatory cytokine receptors [12]. They both map on BTA2 and are separated by a 23 Kb fragment. *CXCR1* and *CXCR2* have been considered as prospective genetic markers for mastitis resistance in dairy cows [13,14]. Deoxycytidine kinase (*DCK*) maps on BTA6 and has a functional role in drug resistance and sensitivity [15]. Toll like receptor 4 (*TLR4*) maps on BTA8 and is associated with the early innate immune response. Specifically, *TLR4* is recognized as the key transmembrane receptor for the detection of gram-negative bacteria [16]. Nucleotide binding oligomerization domain containing 2 (*NOD2*) maps on BTA18 and is a key component in the innate immune system, inducing the activation of proinflammatory signaling pathways [17]. Mannose binding lectin 1 and 2 (*MBL1* and *MBL2*, respectively) map on BTA28 and BTA26, respectively. MBLs are collagenous lectins involved in the innate immune response to various microbial pathogens and are potential candidate gene to mastitis resistance [18]. Lastly, mammary Serum amyloid A3.2 (*M-SAA3.2*) maps on BTA29 and belongs to a superfamily of apolipoproteins expressed in bovine mammary gland as a response to pathogens associated to mastitis [19]. Another candidate gene found in a genomic region close to *DCK*, namely the immunoglobulin J (joining) chain gene (*JCHAIN*), was then included in the re-sequencing to find new possible candidate SNPs: this gene plays an important role in the assembly of polymeric immunoglobulins (dimeric IgA and pentameric IgM) and in their selective transport across epithelial cell layers [20].

The aim of this research was to analyze the genetic variability of these genes in Italian Holstein bulls and to study the effects of their SNPs in relation to resistance to mastitis [21].

## 2. Materials and Methods

### 2.1. Animal Data

The deregressed Estimated Breeding Values for Somatic Cell Score (dEBVs) were obtained by the National Breeding Association (ANAFIJ) for a total of 15,562 Holstein bulls. The dEBVs were calculated as for the genomic national evaluation with the algorithm developed by ANAFIJ. We chose to analyze individuals within the two extreme tails of the dEBVs distribution (±1 SD) of bulls born between 2002 and 2012. Given the 11 years range of bulls’ birth year, dEBVs were expressed as deviation from the mean dEBVs updated in April 2014. Only bulls with a reliability index > 90 and with availability of semen were retained, resulting in the two following subsets: 37 bulls with low dEBVs (group L, <95) and 58 bulls with high dEBVs (group H, >105). Group L dEBVs ranged from 85 to 94 (92.0 ± 2.3, mean ± SD), while group H was composed by bulls with dEBVs values ranging from 106 to 112 (107.9 ± 1.8, mean ± SD). Reliability of the two groups was (mean ± SD) 92.4 ± 0.2 and 92.6 ± 0.2 (L and H, respectively). The mean number of daughters for each bull was 310 ± 74 and 300 ± 57 (L and H, respectively). Semen doses of the selected bulls were obtained from specialized laboratories for semen cryo-conservation.

### 2.2. Genes Data and Re-Sequencing

The DNA of the 95 bulls was re-sequenced in order to detect all the SNPs present in the sequences of the investigated genes. Information were obtained from NCBI database [22], “Gene” section (available from https://www.ncbi.nlm.nih.gov/gene/).

The assembly of the UMD Bovine Genome 3.1.1 was taken as a reference for all the genes (Table 1). The *PTX3* gene mapped on the strand AC_000158.1, corresponding to the sequence of BTA1 in position 1: 111,027,803–111,033,868. The *CXCR1* and *CXCR2* genes both mapped on the strand AC_000159.1, corresponding to the sequence of BTA2 in position 106,936,887–106,938,583 and 106,900,465–106,915,876, respectively. *JCHAIN* was located upstream *DCK* in position 87,759,435–87,768,832 on BTA 6, while *DCK* gene mapped on the strand AC_000163.1 in position 88,049,498–88,077,488. The *TLR4* gene mapped on the strand AC_000165.1, corresponding to the sequence of BTA8 in position 108,828,899–108,839,913. The *NOD2* gene mapped on the strand AC_000175.1, corresponding to the sequence of BTA18 in position 19,177,563–19,212,607. The *MBL1* and *MBL2* genes mapped on the strands AC_000185.1 (BTA28) and AC_000183.1 (BTA26), respectively, in position 35,840,848–35,846,070 and 6,343,615–6,348,912, respectively. Lastly, *M-SAA3.2* gene mapped on the large strand AC_000186.1, corresponding to the sequence of BTA29 in position 26,755,567–26,759,547.

Genomic DNA was extracted from semen by using NucleoSpins Tissue kit (Macherey-Nagel, Düren, Germany). The primers were designed using the Design Studio web application by Illumina^®^ to sequence the entire genes and about 10,000 bp of the upstream regions to search for polymorphisms that could be responsible of the gene expression and be related with resistance to mastitis also in the 5′ UTR regulatory regions. The maximum length of the amplicons for each gene was 450 bp, trying to maximize the coverage of the target region. The primers generated by the software were included in a TruSeq^®^ kit custom amplicon. All the obtained amplicons were sequenced by Next-Generation Sequencing technology on the Illumina^®^ MiSeq platform at the IGA technology Services (Udine, Italy), which also performed the output data processing the variant and genotype call and generated a Variant Call File (VCF) for each gene. Polymorphisms were filtered on the base of locus GQX (genotype quality assuming position, <10,000), GQ (genotype quality, <30.00), R8 (indel repeat length, >8), and MQ (mapping quality, <0.00). Other considered parameters were indel, site conflicts, and read depth. The genotype table was set up in R environment inferring all the identified allelic variants from the VCF files. Allelic frequencies were calculated and mutations with Minor Allele Frequency (MAF) < 0.05 were removed from the dataset.

### 2.3. SNP Analysis

An investigation in the National Center for Biotechnology Information (NCBI, https://www.ncbi.nlm.nih.gov) was carried out on the SNPs with MAF ≥ 0.05 to verify if they were already present in available databases, to define their gene position and, if detected in exon regions, to evaluate their effect on protein translation. The Wilcoxon-Mann-Whitney (WMW) non-parametric test was used to check if there was a difference between the two groups in terms of genotype distribution for all the loci. Subsequently, the generalized heteroscedastic effects regression model (HEM) was used to estimate the contribution of each SNP to the differentiation of the individuals into the two tails of the distribution of the dEBVs [23]. All the SNPs were also simultaneously tested with a multiple gene approach (MG), where association analysis under an additive model was performed considering the phenotype as binary trait (high or low dEBVs) and using the GRAMMAR approach as implemented within the GenABEL package v.1.8 [24] for R [25]. Since the analyzed SNPs were not actually scattered all over the genome, but in 10 selected genes (considering the introduction of *JCHAIN*), polymorphisms with a correlation higher than 0.80 with any others were excluded. Also, the SNPs not in Hardy-Weinberg Equilibrium (HWE) were excluded. Moreover, four individuals having identity by state (IBS) > 0.95, as revealed by the genomic relationship matrix calculated using the entire dataset, were not included in the following analysis. Then, a polygenic analysis was conducted using a genomic kinship matrix based on SNP genotypes to account for relationship between individuals. Residuals from the polygenic analysis were then used as dependent, quantitative variables in single marker, linear regression analyses to test the significance of marker effects.

In order to understand the possible role of the significantly associated SNPs a first check was performed on NCBI database to see if the SNPs fell within regions from which an RNA transcription through RNAseq analysis was obtained so far. Then, short interspersed nuclear elements (SINE), long terminal repeat elements (LTR), and RNA repeats were analyzed using UCSC Genome Browser [26]. Finally, RNA Central was used to search for potential similarity with non-coding RNA [27], while miRbase was used to search for micro-RNA (miRNA) [28].

## 3. Results

The final re-sequenced regions are listed in Table 1. Only for *TLR4*, *NOD2*, *MBL2*, and *MBL1* we obtained sequences for about 10,000 bp upstream the selected genes, whereas for *M-SAA3.2* and *DCK* we obtained reads for about 5600 bp upstream. In the remaining genes, namely *PTX3*, *CXCR1*, *CXCR2*, and *JCHAIN*, we could re-sequence about 1000 bp of the upstream region. For *PTX3* gene we could not obtain the last 1000 bp of the gene. This occurred mainly because some regions were highly repeated while for other regions the software failed in finding specific primers. For the same reasons we could not obtain the 100% coverage, but only a mean gene coverage of 78.6% of the selected regions, with *PTX3* and *MBL2* sequenced with 64% and *CXCR1* and *MBL1* with 88% of coverage respectively. Despite gene selection, other genes are included in the sequenced regions of the bovine genome assembly: *PTX3* gene is included in a region within the ventricular zone expressed PH domain homolog 1 gene (*VEPH1*) and the collectin surfactant protein A (SP-A) gene (*SFPTA1*) is located downstream *MBL1*. Moreover, one significant SNP in *M-SAA3.2* was mapped in an intergenic region that seems to be closer to *SAA4* than to *M-SAA3.2*, about 14,000 bp far from the re-sequencing chosen region. Considering the high similarity of the SAA superfamily, further work is needed to verify if there were mapping errors or we obtained re-sequencing of non-specific products. Therefore, we considered the discovered SNPs as belonging to the selected genes and specified in the annotation column of Table 2 their position referring to other genes.

In the sequenced region the total number of called variants was 25,200, but after quality filtering 2585 SNPs were retained. Out of these 2585 SNPs only 557 had a MAF > 0.05 and therefore were used to analyze their associations with the selected phenotype: 20 SNPs within *PTX3* (BTA1-Supplementary Table S1), three of which located in intron 2 and not found on NCBI SNPs databases (1: 111,030,410, 1: 111,030,420, and 1: 111,030,423); 91 SNPs in *CXCR1* and *CXCR2* (BTA2-Supplementary Table S2), three not found on NCBI SNPs databases (2: 106,913,554, 2: 106,913,717, and 2: 106,913,727) and supposed to lead to missense (V_125_A), synonymous (L_179_L), and missense (V_183_I) mutations, respectively; 76 SNPs within *JCHAIN* and *DCK* (BTA6-Supplementary Table S3), nine not found on NCBI SNPs databases (6: 88,044,038, 6: 88,044,298, 6: 88,044,381, 6: 88,044,420, 6: 88,044,746, 6: 88,064,952, 6: 88,069,416, 6: 88,069,428, and 6: 88,069,437), the first five located in intergenic region and the last four in intron regions; 81 SNPs within *TLR4* (BTA8-Supplementary Table S4); 43 SNPs in *NOD2* (BTA18-Supplementary Table S5); 54 SNPs in *MBL2* (BTA26-Supplementary Table S6); 64 in *MBL1* (BTA28-Supplementary Table S7); 128 SNPs in *M-SAA3.2* (BTA29-Supplementary Table S8). After pruning for correlation and HWE, 182 of these 557 polymorphisms were tested for associations with the MG approach.

WMW test revealed 61 SNPs with a significant different genotype distribution in the two tails of the dEBVs: 12 SNPs out of 20 in *PTX3*, 1 SNP out of the 91 on BTA2 in *CXCR1*, 12 SNPs (three in intron 2 of *JCHAIN*, three in intergenic regions of *DCK*, and six in *DCK* introns) out of 76 on BTA6, six SNPs out of 81 in *TLR4*, 18 SNPs out of 54 in *MBL2*, 3 SNPs out of 64 in *MBL1*, four SNPs out of 128 in M-SSA3.2. Both the HEM and the MG analysis showed nine SNPs significantly associated with the phenotype each. Since two SNPs obtained by HEM and one SNP obtained by MG approach had no significant differences in genotype distribution by WMW, the total number of SNPs included in Table 2 is 64. The SNPs position refers to the UMD Bovine Genome 3.1.1 assembly and, where available, the SNPs rs are reported together with the description of the type of variant, the level of significance of the three tests and the effect of the SNP for HEM and MG approaches. The HEM approach revealed significant SNPs in *DCK* (4), *NOD2* (2), *MBL2* (2), *MBL1* (1), whereas the MG approach revealed significant SNPs in *CXCR1* (1), *DCK* (3), *NOD2* (3), and *M-SAA3.2* (2). None of the SNPs in *PTX3*, *CXCR2*, *JCHAIN* and *TLR4* resulted significantly associated with the SCS levels in the analyzed population. Nevertheless, one SNP in *PTX3* mapped within 3′ UTR and others two corresponded to the non-synonymous mutations responsible for the aminoacidic exchanges D_341_E and E_347_K, therefore they could lead to changes in the expressed protein and be matter of interest although they were not significantly associated with the SCS levels in the analyzed population.

### 3.1. CXCR1

The only SNP showing a different genotype distribution in L and H groups by WNW test, rs109694601, also resulted significantly associated with SCS levels by MG approach. This SNP, located within intron region in *CXCR1*, is mapped 24 pb upstream a Mammalian-wide Interspersed Repeats (MIR) element of the MIR3 subfamily. MIRs represent an ancient family of tRNA-derived Short INterspersed Elements (SINEs) found in all mammalian genomes, whose core region may serve some general function, although they have ceased to amplify by retro-transposition. Despite their massive presence in mammalian genomes, their contribution to the transcriptome is still largely unexplored even in humans, and in particular, elements controlling their transcription have never been systematically studied [29], thus we cannot even hypothesize a role of this SNP yet.

### 3.2. DCK

None of the SNPs located in *JCHAIN* showed significant associations with the considered phenotype, whereas a total of five SNPs in *DCK* were found significantly associated with dEBVs levels with HEM (four SNPs) and MG (three SNPs) approach. It has to be noted that despite the pruning of the high correlated SNPs and the removal of four individuals because of high IBS by MG approach, which could lead to slightly different results with respect to HEM, the two SNPs not found on NCBI databases (6: 88,044,420 and 6: 88,069,428) resulted significant with both HEM and MG approach (Table 2). The intergenic region comprising the SNP at position 88,044,420 and SNP rs1115177107 includes a transcribed RNA, as shown by NCBI database, and could therefore have some not yet described regulatory effects. In *DCK* intron 1, where two significant SNPs (rs43472176 and rs43472180) were described, two ncRNA-like sequences were also found. Moreover, in-between rs43472177 and rs43472180 we found a sequence showing high homology with BTA-mir-2452, a miRNA found expressed upon induced viral infection [30], indicating that it should have a role in the immune response.

### 3.3. NOD2

Four out of the six SNPs with significant genotype distribution in H and L groups, were also found significantly associated with dEBVs levels using MG (rs109352180, rs209159307, and rs110918103) and HEM (rs110918103, and rs210362219) approaches. As for *DCK* also for *NOD2* there was at least one SNP in common between the two approaches (rs110918103). The intergenic region comprising rs109352180 shows the presence of transcribed RNA and could therefore have regulatory effects. Moreover, rs109352180 flanks a region partially aligning with three human miRNAs and four bovine lncRNAs, three of which are precursors of miR-185, a miRNA that could influence the expression of different genes [31,32]. The region is also rich in SINEs, whereas rs210362219 is in a sequence aligning with 3 bovine lncRNA and one human miRNA (68% identity).

### 3.4. MBL2

None of the 18 SNPs that could be of interest on the basis of WMW test resulted significantly associated with SCS levels by MG approach, neither the three mapped within exon regions (rs209940244, rs210820536, rs463533307), all synonymous mutations, nor the three in proximity of 5′ UTR (Table 2). With HEM approach two SNPs resulted significantly associated with the phenotype: rs110884426, mapped in the intergenic region and rs136687134, located about 1000 bp downstream the 3′ UTR. Both SNPs are found in a transcribed RNA, as shown by NCBI database. Moreover, rs136687134 is included in a region of Long INterspersed Elements (LINEs), transposable elements that take a large proportion of eukaryotic genomes, once regarded as nonfunctional sequences, and now considered to play pivotal roles in gene regulation [33,34].

### 3.5. MBL1

None of the 3 SNPs that could be of interest on the basis of WMW test resulted significantly associated with SCS levels by MG approach, whereas rs211629255, the SNP that falls into intron 2 of *SFPTA1*, resulted significant by the HEM approach (Table 2).

### 3.6. M-SAA3.2

The WMW test showed that genotypes distribution in the two sample groups was significantly different for three SNPs, namely rs136687125, rs137746604, and rs210417381. Of the three SNPs, only rs210417381, with also rs42175273, was found to be significantly associated with the phenotype in the MG approach, (Table 2). It has to be considered that the three SNPs located near the 5′ UTR of *M-SAA3.2* have a mean correlation of about 0.77 and were therefore all included in the MG analysis, but due to the high correlation among them it is difficult to define which SNPs has actually an effect or if they are all involved in the response to mastitis resistance. The 3 SNPs are included in an RNA transcribed region containing 4 ncRNAs and also a long terminal repeated (LTR) element that should belong to endogenous retrovirus KERVK family. There are evidences that LTR sequences derived from distantly related endogenous retroviruses (ERVs) act as regulatory sequences for many host genes in a wide range of cell types throughout mammalian evolution [35,36]. Re-activation of ERVs is often associated with inflammatory diseases, thus the region could actually be related with mastitis resistance/susceptibility.

## 4. Discussion

Starting from the knowledge of the correlation between SCC and mastitis [37], the aim of this study was to analyze the genetic variability of candidate genes and to investigate the association of the identified SNPs with SCS EBVs. As reported by Koeck et al. [38], SCS EBVs are a valuable alternative predictor for mastitis resistance. Candidate genes selected for this study were already known in literature to be related to immune response to mastitis infections [11,12,13,14,15,16,17,18,19]. To maximize the effects of the different SNPs, bulls to be sequenced were selected between the low and high tails of distribution for SCS. We chose to use the selective genotyping approach: only individuals from the high and low extremes of the trait distribution are selected for genotyping, reducing genotyping work and costs maintaining nearly equivalent efficiency to complete genotyping [39,40].

An overall total of 64 SNPs showed significantly different genotype distributions in the H and L groups or were identified as significantly associated with dEBVs for SCS. Among these SNPs, 15 resulted significantly associated with HM or MG approach and 3 of them with both approaches. The main difference between the two approaches used in this study lies in MG that takes into account the genomic relationship matrix and the correlation between SNPs. Indeed, when correlation is considered, several SNPs associations could actually belong to correlated SNPs that were excluded from the analysis rather than to the reported SNP. Moreover, with the MG approach, given the general small allele substitution effect when considering simultaneously SNPs in different genes, and using a correction for the genomic relationship, a low number of significant SNPs is expected. The remaining SNPs should be, therefore, the ones where the association is stronger. Of the 15 SNPs identified by both approaches, the most significant were located inside intronic regions within *DCK* (rs43472176 and one at position 6: 88,069,428 not previously available on NCBI SNPs databases) and *NOD2* (rs110918103). The newly discovered SNP at position 6: 88,069,428, together with rs110918103, are two of the three SNPs resulted significantly associated with both HM and MG approach. No significantly associated SNPs were found in exon regions, while eight SNPs were located in the intergenic regions and could possibly be related to distal regulatory element. Six genes (*CXCR1*, *DCK*, *NOD2*, *MBL2*, *MBL1* and *M-SAA3.2*) included SNPs that were identified as significantly associated by HEM and/or MG and that are known to be key components in the immune system. As reported by Siebert et al. [41], several studies identified different SNPs in gene associated with mastitis and, furthermore, with SCS. Similarly, *DCK* gene was suggested as candidate gene associated with mastitis [42]. On the other hand, the direct association between mastitis and *MBL1*, *MBL2*, *NOD2* and *M-SAA3.2* genes has not been fully explored so far. Nevertheless Wang et al. [43], using a GWAS approach found a significant SNP in *SAA2* gene, indicating an important role of the superfamily of these apolipoproteins. These results suggest that, just like *CXCR1* and *DCK* genes, also *MBL1*, *MBL2*, *NOD2* and *M-SAA3.2* genes could be eligible as candidate genes for genetic selection of mastitis resistant cows. Many studies demonstrated that SNPs associated with diseases are mainly located in non-coding regions, making it difficult to link them to specific biological pathways. A recent study, in which a genotyping-by-sequencing approach was used to find novel SNPs associated with milk traits, showed that the majority of identified SNPs were located within intergenic regions (69%), followed by intronic regions (25%), with only 3.46% of SNPs being coding variants [44]. Moreover, different studies found that conserved non-coding regions in introns and near genes show large allelic frequency shifts, similar in magnitude to missense variations, suggesting that they are critical for gene function regulation and evolution in many species [45,46]. Most of the significant SNPs detected in our investigation are located inside or in proximity to complete or partial lncRNA-like or miRNA-like sequences, or to repeated regions containing SINEs or LINEs, and it is now established that both intronic and LINE/SINEs repeats could lead to transcriptional regulation of the affected genes [47]. Thus, although the role of some of these elements, especially in the bovine species, has still to be verified, and further studies are needed to better understand the role of these SNPs, the results obtained here are a starting point. Contrary to the findings of Welderufael et al. [48], no SNPs were identified as significantly associated with dEBVs level within *PTX3* gene in our population. Similarly, no significant SNPs were detected within candidate genes *TLR4* [49].

## 5. Conclusions

In this study, next generation sequencing technology was used to discover new SNPs in candidate genes related to mastitis resistance and to identify those associated with dEBVs for SCS. We analyzed the genetic variability of several SNPs found in candidate genes and identified 15 that are associated with the phenotype. Two of them maps within *DCK* gene and were not previously available on NCBI database, whereas other SNPs strengthen the role of *CXCR1*, *NOD2*, *MBL1*, *MBL2*, and *M-SAA3.2* as candidate genes. These results suggest that the possibility to use SNPs as markers for genetic selection of mastitis resistant cattle is plausible. 

## Figures and Tables

**Table 1:** Position of the selected genes on UMD bovine genome 3.1.1, target region selected for resequencing, genes’ upstream and downstream regions sequenced, and gene sequencing coverage (COV).

GENE (CHR)	Gene Position		ReSeq Region		Upstream	Downstream	COV
*PTX3* (BTA1) ^1^	111,027,803	111,033,868	111,026,949	111,032,707	854	−1161	64%
*CXCR1* (BTA2)	106,936,878	106,938,583	106,935,752	106,942,024	1126	3441	88%
*CXCR2* (BTA2)	106,900,475	106,915,876	106,899,301	106,917,188	1174	1312	73%
*JCHAIN* (BTA6)	87,759,435	87,768,832	87,758,532	87,770,133	903	1301	85%
*DCK* (BTA6)	88,049,498	88,077,488	88,043,812	88,077,721	5686	233	78%
*TLR4* (BTA8)	108,828,899	108,839,913	108,818,057	108,841,671	10,842	1758	81%
*NOD2* (BTA18)	19,177,563	19,212,607	19,166,798	19,213,798	10,765	1191	83%
*MBL2* (BTA26)	6,343,615	6,348,912	6,332,528	6,349,772	11,087	860	64%
*MBL1* (BTA28) ^1^	35,840,848	35,846,070	35,839,722	35,856,132	10,062	1126	88%
*M-SAA3.2* (BTA29)	26,755,567	26,759,547	26,749,896	26,760,832	5671	1285	82%

^1^ reverse oriented gene.

**Table 2:** SNPs resulted significantly different in frequency in the two tail of the deregressed EBVs for SCS as calculated with Wilcoxon-Mann-Whitney (WMW) test. The SNP resulted significantly associated with the phenotype with the Heteroscedastic Effects regression Mode (HEM) and the multiple gene approach (MG) are also reported together with the SNP effect.

Gene	Chr	Position	rs	Allele	Annotation	WMW *p* ^5^	HEM	MG *p* ^5^
*PTX3* ^1^	1	111,028,365	rs378618073	G/T	3′ UTR	***	ns	ns
		111,028,516	rs208223246	C/T	exon 3 (E_347_K)	***	ns	ns
		111,028,532	rs43263271	A/C	exon 3 (D_341_E)	*	ns	ns
		111,030,195	rs207576885	A/T	intron 2	***	ns	ns
		111,030,376	rs210764862	G/A	intron 2	***	ns	ns
		111,030,399	rs381383694	A/C	intron 2	***	ns	ns
		111,030,410	NA ^2^	A/G	intron 2	***	ns	ns
		111,030,413	rs208776659	C/G	intron 2	***	ns	ns
		111,030,423	NA	T/C	intron 2	***	ns	ns
		111,030,988	rs381920578	C/T	intron 2	**	ns	ns
		111,031,525	rs207709330	T/A	intron 2	*	ns	ns
		111,031,892	rs43263268	A/C	intron 2	***	ns	ns
*CXCR1*	2	106,939,924	rs109694601	G/A	intron 1	**	ns	−0.159 (A) *
*JCHAIN*	6	87,762,375	rs110597692	C/G	intron 2	*	ns	ns
		87,762,415	rs110854643	G/A	intron 2	*	ns	ns
		87,764,301	rs382005122	C/T	intron 2	*	ns	ns
*DCK*	6	88,043,981	rs1115177107	G/A	intergenic	*	ns	ns
		88,044,420	NA	G/T	intergenic	*	0.432 (T) *	0.125 (T) *
		88,048,414	rs137327740	C/G	−1.084 5′ UTR	*	ns	ns
		88,054,256	rs43472176	T/C	intron 1	***	ns	−0.205 (C) **
		88,054,483	rs43472177	C/T	intron 1	***	ns	ns
		88,055,035	rs43472180	T/C	intron 1	***	−0.451 (C) *	ns
		88,059,177	rs379452380	-/T	intron 2	*	ns	ns
		88,069,402	rs452449360	T/C	intron 4	*	−0.418 (C) *	ns
		88,069,428	NA	T/A	intron 4	**	−0.699 (T) *	−0.186 (T) **
*TLR4*	8	108,822,089	rs43578057	T/C	intergenic	*	ns	ns
		108,822,381	rs43578059	A/C	intergenic	*	ns	ns
		108,822,406	rs43578060	C/T	intergenic	*	ns	ns
		108,822,446	rs43578061	G/A	intergenic	*	ns	ns
		108,822,466	rs43578062	T/A	intergenic	*	ns	ns
		108,822,579	rs43578063	T/A	intergenic	*	ns	ns
*NOD2*	18	19,168,779	rs111017375	A/C	intergenic	*	ns	ns
		19,168,902	rs109352180	T/C	intergenic	**	ns	0.162 (C) *
		19,186,138	rs209462767	G/A	splice exon 4	*	ns	ns
		19,205,258	rs209159307	G/A	intron 11	*	ns	−0.195 (A) *
		19,210,095	rs110918103	T/A	intron 12	**	0.303 (A) *	0.159 (A) **
		19,210,136	rs210362219	G/A	intron 12	**	0.303 (A) *	ns
*MBL2*	26	6,332,839	rs110884426	C/T	intergenic	ns	−0.421 (T) *	ns
		6,332,909	rs208727559	T/A	intergenic	*	ns	ns
		6,332,959	rs209975765	C/T	intergenic	*	ns	ns
		6,334,846	rs520561418	C/T	intergenic	*	ns	ns
		6,335,302	rs442274187	G/T	intergenic	*	ns	ns
		6,341,013	rs380597712	A/C	intergenic	*	ns	ns
		6,341,147	rs465968175	T/C	intergenic	*	ns	ns
		6,342,415	rs459506838	TTAA/-	−1200 5′ UTR	*	ns	ns
		6,342,906	rs482417200	T/A	−709 5′ UTR	*	ns	ns
		6,343,309	rs798205710	C/T	−306 5′ UTR	*	ns	ns
		6,343,517	rs384805952	T/C	−98 5′ UTR	*	ns	ns
		6,343,820	rs438573157	G/T	intron 1	*	ns	ns
		6,344,627	rs436853860	A/G	intron 1	*	ns	ns
		6,344,678	rs455369386	C/T	intron1	*	ns	ns
		6,344,920	rs209940244	G/A	exon 2 (P_42_P)	*	ns	ns
		6,344,929	rs210820536	T/C	exon 2 (N_45_N)	*	ns	ns
		6,345,350	rs438686412	A/G	intron 2	*	ns	ns
		6,345,401	rs463533307	C/T	exon 3 (P_73_P)	*	ns	ns
		6,345,502	rs475632625	C/G	intron 3	*	ns	ns
		6,349,735	rs136687134	A/C	+823 3′ UTR	ns	0.242 (C) *	ns
*MBL1* ^1^	28	35,842,351	rs208247354	G/A	intron 3	*	ns	ns
		35,844,679	rs208491630	G/T	intron 1	*	ns	ns
		35,852,741	rs211629255	C/T	intron 2 *SFPTA1* ^3^	**	0.323 (T) *	ns
*M-SAA3.2*	29	26,741,245	rs42175273	A/T	intergenic *SAA4* ^4^	ns	ns	−0.126 (T) *
		26,755,302	rs136687125	T/C	−265 5′ UTR	*	ns	ns
		26,755,410	rs137746604	G/A	−157 5′ UTR	*	ns	ns
		26,755,477	rs210417381	T/C	−90 5′ UTR	*	ns	−0.181 (C) *

^1^ reverse oriented gene; ^2^ NA = not available; ^3^ the SNP falls in intron 2 of the collectin surfactant protein A gene *SFPTA1*; ^4^ the SNP falls in an intergenic region mapped near *SAA4* gene; ^5^ *p*-values: * *p* < 0.05, ** *p* < 0.01, *** *p* < 0.001, ns = not significant.

## Data Availability

Data is contained within the article or as supplementary material.

## Supplementary Materials

The following are available online at https://www.mdpi.com/2076-2615/11/2/366/s1, Table S1: 20 SNPs found within *PTX3* gene, Table S2: 91 SNPs found within *CXCR1* and *CXCR2* genes, Table S3: 76 SNPs found within *DCK* gene, Table S4: 81 SNPs found within *TLR4* gene, Table S5: 43 SNPs found within *NOD2* gene, Table S6: 54 SNPs found within *MBL2* gene, Table S7: 64 SNPs found within *MBL1* gene, Table S8: 128 SNPs found within SAA3 gene.
